# Health risks of climate change: An assessment of uncertainties and its implications for adaptation policies

**DOI:** 10.1186/1476-069X-11-67

**Published:** 2012-09-19

**Authors:** J Arjan Wardekker, Arie de Jong, Leendert van Bree, Wim C Turkenburg, Jeroen P van der Sluijs

**Affiliations:** 1 Department of Innovation, Environmental and Energy Sciences, Copernicus Institute of Sustainable Development, Utrecht University, Budapestlaan 6, 3584 CD, Utrecht, Netherlands; 2 Netherlands Environmental Assessment Agency (PBL), P.O. Box 30314, 2500 GH, The Hague, Netherlands

**Keywords:** Climate change and health, Uncertainty, Knowledge quality assessment, Expert elicitation, Climate adaptation policy

## Abstract

**Background:**

Projections of health risks of climate change are surrounded with uncertainties in knowledge. Understanding of these uncertainties will help the selection of appropriate adaptation policies.

**Methods:**

We made an inventory of conceivable health impacts of climate change, explored the type and level of uncertainty for each impact, and discussed its implications for adaptation policy. A questionnaire-based expert elicitation was performed using an ordinal scoring scale. Experts were asked to indicate the level of precision with which health risks can be estimated, given the present state of knowledge. We assessed the individual scores, the expertise-weighted descriptive statistics, and the argumentation given for each score. Suggestions were made for how dealing with uncertainties could be taken into account in climate change adaptation policy strategies.

**Results:**

The results showed that the direction of change could be indicated for most anticipated health effects. For several potential effects, too little knowledge exists to indicate whether any impact will occur, or whether the impact will be positive or negative. For several effects, rough ‘order-of-magnitude’ estimates were considered possible. Factors limiting health impact quantification include: lack of data, multi-causality, unknown impacts considering a high-quality health system, complex cause-effect relations leading to multi-directional impacts, possible changes of present-day response-relations, and difficulties in predicting local climate impacts. Participants considered heat-related mortality and non-endemic vector-borne diseases particularly relevant for climate change adaptation.

**Conclusions:**

For possible climate related health impacts characterised by ignorance, adaptation policies that focus on enhancing the health system’s and society’s capability of dealing with possible future changes, uncertainties and surprises (e.g. through resilience, flexibility, and adaptive capacity) are most appropriate. For climate related health effects for which rough risk estimates are available, ‘robust decision-making’ is recommended. For health effects with limited societal and policy relevance, we recommend focusing on no-regret measures. For highly relevant health effects, precautionary measures can be considered. This study indicated that analysing and characterising uncertainty by means of a typology can be a very useful approach for selection and prioritization of preferred adaptation policies to reduce future climate related health risks.

## Background

Climate change is projected to have wide-ranging effects on physical, ecological and societal systems. Conceivable health-related impacts include changes in temperature-related mortality, malnutrition, infectious diseases, environmental quality, natural disasters, and societal stability [1-3]. The health dimensions of climate change are gaining increasing interest in both the scientific and policy communities as societally relevant impacts. In the Netherlands, for instance, scientists and municipalities have recently conducted various studies on urban heat stress and heat island effects, and several impact and adaptation assessments have looked at the general topic of climate change and health. At the European level, both the European Commission and scientific organisations, such as the European Environment Agency, the European Centre for Disease Prevention and Control, and WHO Europe, have taken interest.

Assessments of climate change impacts involve uncertainty in every step of the analysis, from assumptions about socio-economic developments (leading up to emission scenarios), their implications for future global and local climates and environment (as assessed using e.g. various models and their associated assumptions), to assessing the impacts on society (which is itself non-static and subject to uncertain changes) [4,5]. Consequently, these uncertainties add-up in a ‘cascade’ of uncertainty. Health risks arise from the interaction of uncertain future climatic changes with complex ecological, physical, and socio-economic systems, which are simultaneously affected by numerous other changes, e.g. globalisation, demographic changes, and changes in land use, nutrition, health care quality. Policymaking on adaptation to health risks of climate change thus faces substantial uncertainty.

Health impact assessments of climate change frequently indicate uncertainties. Examples include: 95 %-confidence intervals for exposure-response relationships (e.g. temperature-mortality), geographical and temporal variability, ranges of published climate scenarios, co-existence of equally plausible model structures, differences between impact assessments due to different underlying assumptions, limited available empirical data, questions regarding the applicability of short-term historical relationships to long-term projections, biases, multi-factorial causal webs, confounders, non-linear responses, and various knowledge gaps [2,6-11]. Such uncertainties extend far beyond confidence intervals and similar metrics, which represent only statistical uncertainties and may not include all relevant (or even key) factors and parameters [12]. Deeper levels of uncertainty limit the reliability of health risk assessments of climate change (cf. [13]).

While there are many uncertainties, this does not mean that climate change adaptation cannot meaningfully take place [14]. Similarly, impact assessment is still possible; if predictions or projections are not feasible or sensible, one might still be able to perform simple order-of-magnitude or bounding analyses [15]. Some approaches to adaptation can, however, handle certain types and levels of uncertainty better than others. Dessai and Van der Sluijs [5] present a framework in which they relate the suitability of various adaptation approaches to three levels of uncertainty: statistical uncertainty, scenario uncertainty, and recognized ignorance and surprise^a^. See the Additional file 1: Table S1. For example, quantitative (health) risk approaches handle statistical uncertainties quite well, but fail to tackle other types of uncertainty. Resilience-oriented approaches, on the other hand, can cope well with ignorance and surprises, but are less appropriate when statistical uncertainty prevails. Thus, the level and nature of uncertainty have important implications for selecting appropriate adaptation approaches and for policy choices regarding their implementation.

Uncertainty is frequently discussed in the literature on climate and health, but this information is spread over multiple research fields. Synthesis documents do provide information on major sources of uncertainty and knowledge gaps, but not on the *level* of uncertainty, nor on differences among effects/topics (i.e. are we more ignorant about one effect than another?). We applied expert elicitation to explore this information in detail. This paper investigates the level of uncertainty for various conceivable health impacts of climate change, using the ‘Level of Precision’ scale developed by Risbey and Kandlikar [16,17] (table 1); ranging from ignorance to probabilistic estimates. This scale allows for an ordinal comparison of the levels of uncertainty between health effects. Experts participating in this elicitation were asked to assess the level of precision with which they would be able to estimate the magnitude of particular health risks due to climate change for the Netherlands (see Methods section). Policy implications of this uncertainty assessment will be discussed based on (and expanding on) the framework proposed by Dessai and Van der Sluijs [5].

**Table 1:** **Level of Precision scale (based on**[16,17]**)**

**Score:**	**Label:**	**Description:**
1	Effective ignorance	Knowledge of the factors that govern this effect is so weak that we are effectively ignorant.
2	Ambiguous sign or trend	Some effect is expected, but its sign or trend is not clear. There are plausible arguments either direction (effect could be positive, could be negative; could increase or decrease).
3	Expected sign or trend	It is clear what the sign and trend of the effect will be. However, there is no plausible or reliable information on how strong it will be.
4	Order of magnitude	It is possible to give a rough indication of the magnitude of the effect, a qualitative scoring (e.g. 1–10 scale), or a rough comparison with other effects.
5	Bounds	It is possible to estimate the bounds for the distribution of the effect, e.g. its 5/95 percentiles (effect is only 5 % likely to be more than … and only 5 % likely to be less than …). However, the shape of the distribution, or best-guess estimates, cannot be provided.
6	Full probability density function	It is possible to provide a full probability density function; the bounds as well as the shape of the distribution.
N/A	Don't know / no answer	

## Methods

### Setup

A formal expert elicitation was performed to assess the levels of uncertainty associated with conceivable health impacts of climate change in the Netherlands, and their implications for climate change adaptation. Expert elicitation is a structured approach of consulting experts on a subject where there is insufficient knowledge in the published literature. It seeks to make explicit and synthesise the published and unpublished knowledge and insight of experts (e.g. [18-20]), including limitations, strengths and weaknesses of published knowledge and available data. Multiple steps can be discerned (see Additional file 1). Literature analysis, inventorying relevant subtopics and uncertainties, provides the basis for the elicitation’s design and scope. A list of relevant health effects was compiled based on recent Dutch impact assessments [8,9,21]. The draft list included all health-relevant effects that were connected with climate change and climate variables in the available impact assessments. This included e.g. flooding-related impacts, but excluded very indirect effects such as through climate change impacts on biodiversity, food availability, and global social issues (e.g. wars, migration). The effects were grouped in themes, corresponding with different areas of expertise, to allow experts to select the sections of the questionnaire that they had sufficient expertise to answer. Different effects were sometimes aggregated, e.g. ‘pollen types, abundance, and allergenicity’, when the effects were similar and expected not to differ in level of precision rating. Several experts with a good overview of the field were consulted to review the list, the themes, and the aggregations. Table 2 presents the final list.

Knol et al. [20] review methods and approaches to expert elicitation such as workshops/panels, face-to-face interviews, or questionnaires. Our study used an online, in-depth questionnaire, because of the broadness and fragmented nature of the field of ‘climate change and health’, and preference for a standardised format.

The study focused on the Netherlands to prevent biases due to possible local/regional differences in predictability and uncertainty. Additionally, the outcomes may provide initial input concerning impacts and adaptation under uncertainty for national-level assessments, such as the further development of a ‘roadmap to a climate-proof Netherlands’ [22].

Participants were given the opportunity to comment on this paper before it was submitted.

### Expert selection

External experts with good overviews of the networks of Dutch, Belgian, and European researchers were provided with the questionnaire and background information, and were asked to nominate experts with sufficient relevant knowledge to assess the questions posed (explicitly on climate & health, uncertainties, and adaptation). The resulting list was invited; the invitation included a suggestion to forward it to additional relevant experts. The list included scientists and knowledgeable professionals. A total of 21 experts participated (see Additional file 1). Responses were submitted during June-September 2009. Individual quantitative questions were answered by 8–17 experts each (mean: 12.6). This is well within the range that is usually aimed for in expert elicitations; 6–12 participants [20,23].

Participants were asked to indicate their areas of expertise, allowing a distinction between generalists and subject-matter experts on specific questions. They were instructed to answer only those questions that they considered themselves capable of assessing. All health themes were assessed by subject-matter experts; 1–5 (mean: 3.1) per theme. Expertises ‘adaptation’ and ‘health and adaptation’ were represented by 8 and 6 subject-matter experts respectively. Expertises were used in weighting and interpreting the results, particularly to uncover any discrepancies between generalist and subject-matter expert scorings and arguments.

### Protocol and analysis

The questionnaire (see Additional file 1) used both quantitative and qualitative questions, often using a scoring scale (Level of Precision, table 1) or rank-order of a health effect followed by argumentation. Argumentations were important for understanding and analyzing the scores, and to stimulate active reflection on the available evidence by the participant in the process of scoring. Responses to qualitative questions were analysed for lines of argument, and for similarities, differences, biases and consistency of these (within and between questions and scores).

The main part of the questionnaire investigated the level of uncertainty associated with the various health impacts. The experts were asked: “Regarding the following specific health issues, with what level of precision would you be able to estimate the magnitude of the health risk for the Netherlands (due to climate change)? Assume you would be given some time to review the relevant literature, before you would make the effect estimate.” The question did not consider a single climate scenario (although respondents may have interpreted it as such). As different experts may have different views on which factors are relevant to answer the question above (e.g. only climatic or also non-climatic, such as the state of the healthcare system), they were left free to decide which factors to include in their assessment. We explicitly asked respondents to provide a clear argumentation for each score given: their reasons for assigning scores are as valuable as the scores themselves. When an expert answered with a range, his vote was equally divided over these scores. Group scores were created using the weighted median and interquartile range of individual scores. Subject-matter experts were given double weight.

The questionnaire’s second part focused on policy implications. Participants were asked to indicate and rank the five health effects they considered most ‘relevant’ for Dutch climate adaptation policy in view of health. Respondents were asked to take ‘relevance’ in a broad sense, including health, economic and political implications.^b^ As such, this measure represents the societal salience of the effect. The answers to connected open-ended questions concerning adaptation options are discussed in the Additional file 1. Final scores were created per effect; assigning 5 points for each time selected as most relevant, 4 points for second-most relevant, et cetera. Final scores were grouped into four classes (I: 0 points, II: 1–10 points, III: 11–20 points, IV: ≥21 points) to reduce the impact of an unwarranted level of resolution, considering the number of respondents to this question (n=16) and of potential bias of experts towards rating their own fields as particularly relevant.

## Results

A list of 33 potential health impacts of climate change was identified and grouped into eight health themes. Level of precision scores were elicited from 21 participating experts (see Methods section). table 2 lists the scores. The final section discusses the relevance of health effects for adaptation.

**Table 2:** Scoring for the ‘Level of Precision’ with which climate change-related health risks for the Netherlands can be assessed

**Health effect**	**Level of Precision**^**a**^							
	**Frequency/score**^**b**^						**Median**^**c**^	**Inter quartile**^**c**^
	**1**	**2**	**3**	**4**	**5**	**6**		
**Temperature**								
1. Heat-related mortality				9 (2)	3 (1)	2	4	4-5
2. Heat-related cardiovascular problems		½	9½ (2)	3	1 (1)	1	3	3-4
3. Heat-related respiratory problems			11 (2)	3	2 (1)		3	3-4
4. Heat-related stress and sleep disturbance		1 (1)	8 (2)	5			3	3-4
5. Cold-related mortality		3 (1)	2	7 (1)	2 (1)	1	4	3-4
6. Cold-related diseases	1 (1)	2	7 (1)	3	2 (1)		3	3-4
7. Drought-related exposure to contaminants		5 (1)	6 (1)	2			3	2-3
8. Shortages of drinking water		3 (1)	3	5 (1)		1	3½	2¼-4
9. Dehydration		5 (1)	5 (1)	3	1 (1)		3	2-4
**Allergies**								
10. Asthma	1	4	7 (4)	1			3	2-3
11. Allergic eczema	1	5 (1)	3 (1)				2	2-3
12. Hay fever: duration of pollen season			10 (4)	2 (1)	3		3	3-3½
13. Hay fever: pollen types, abundance and allergenicity		1	10 (5)	2	2		3	3
**Pests**								
14. Wasps	1	3 (1)	2 (1)	1	1		2½	2-3
15. Oak processionary caterpillar			1	8 (2)	2		4	4
**Vector-borne diseases**								
16. Native vector-borne diseases		7 (3)	4 (1)	5 (1)	1		3	2-4
17. Incidents of non-native vector-borne diseases	1¼ (¼)	5¼ (2¼)	5¼ (2¼)	4¼ (¼)			3	2-3
18. Epidemics of non-native vector-borne diseases	1¼ (¼)	6¾ (2¼)	4¾ (1¼)	2¼ (¼)			2½	2-3
**Food/water-borne diseases**								
19. Food poisoning	1	1	6	5 (1)			3	3-4
20. Legionnaires Disease		2	7	2 (1)	1		3	3-4
21. Contamination of swimming/recreation water			4	7 (1)	1		4	3-4
**Air quality-related**								
22. Respiratory problems due to ground-level O_3_		1½	4½	4 (2)	2 (1)		4	3-4
23. Respiratory problems due to PM		1½	3½	3 (2)	2 (1)		4	3-4
24. Air quality-related cardiovascular problems		2	3	3 (2)	2 (1)		4	3-4
**Flooding/storm**								
25. Flood-related mortality		4	2	2½ (½)	3½ (1½)		4	2¼-4⅞
26. Flood-related infectious diseases		5 (1)	5 (1)	1			3	2-3
27. Flood-related exposure to dangerous substances and contaminants	1	5 (2)	3	2			2	2-3
28. Flood-related respiratory problems	1	3	5 (1)	1 (1)	1		3	2-3
29. Flood-related mental health problems		2	7 (2)	1			3	3
30. Storm-related mortality and injury		3	3 (2)	4	1		3	3-4
**UV-related**								
31. Cataract	1 (1)	3	1	1	3 (2)		3½	2-5
32. Skin cancer	1 (1)	3	2	2	4 (2)		4	2-5
33. Weakening of the immune system	2 (1)	3	1	2 (1)	1 (1)		2½	1¾-4

^a^See table 1 for scoring scale. ^b^Total experts/score; subject-matter experts are indicated between parentheses. ^c^Weighted.

### Temperature

Changing temperatures may affect premature mortality and morbidity through effects on cardiovascular and respiratory diseases, or various indirect effects (e.g. drought-related increase pollutant-concentrations, dehydration). In terms of achievable precision of impact assessment, heat-related mortality received the highest-score in this study: median 4 (interquartile (i.q.): 4–5). Cold-related mortality scored 4 (i.q.: 3–4).

Regarding heat-related and cold-related mortality, respondents noted that much data, experience, and literature is available. One generalist, scoring heat-related mortality at ‘full PDF’, suggested that it shouldn’t be difficult to “tune a model for mortality surveillance or expected mortality”. Most experts, however, indicated that projections based on present-day epidemiological evidence are limited by:

limited data for the Netherlands (cf. [24]; temperature-mortality relation is based on only six heat waves and five cold spells),

confounders and interactions with other changes (e.g. socio-economic, air quality, demographics, harvesting effect),

possible changes of the response function (e.g. physiological adaptation, behavioural changes, changes in building practices such as availability of air conditioning),

limited knowledge on why response functions differ across places,

difficulties in assessing future heat wave intensity, duration, and frequency,

limited knowledge on the (biophysical) ‘why’ of heat-related mortality and precise metrics which are causally linked to the effect.

One subject-matter expert scored cold-related mortality at ‘ambiguous sign/trend’, suggesting that it could increase, rather than decrease, under some climate scenarios and assumptions on autonomous adaptation, although only one study [25] has demonstrated this. The cited study does, however, provide order-of-magnitude estimates of these cases.

For temperature-related diseases, most participants indicated that the effects of (changing) temperature(s) were well-documented in literature, particularly for the elderly, but data (in general and Netherlands-specific) is lacking to make reliable order-of-magnitude assessments. For respiratory problems, the interaction with hay fever and air quality effects was mentioned as confounders. Arguments for higher scores referred only to the availability of literature and epidemiological data, such as on the 2003 European heat wave. For cold-related diseases, one subject-matter expert (scoring 1) noted that it is still unclear why influenza is a seasonal disease.

Regarding indirect effects (effects 7–9 in table 2), many respondents pointed to a lack of data, although there are some indications that climate change may affect these issues. Arguments for low scores suggested that it was unclear whether health impacts would take place, considering the well-prepared societal care system. Arguments for high scores indicated existing reports/modelling and the availability of short-term abatement options that would limit impacts (providing a constraint for the estimate).

### Allergies

An increasing growing/blooming season, and changes in relative humidity may have implications for e.g. (aero) allergens, particularly pollen, and house dust mite allergen. This would affect health through changes in asthma, allergic eczema and hay fever. Allergic eczema scored 2 (i.q.: 2–3); asthma 3 (i.q.: 2–3) and hay fever-effects 3 (i.q.: 3–3 and 3-3½).

Regarding asthma and allergic eczema, subject-matter experts indicated that negative effects can be expected, due to the expected impacts of climate change on hay fever. However, asthma is a highly multi-factorial/multi-causal disease and there is a lack of data, particularly for the Netherlands. The magnitude of health impacts under various climate scenarios was deemed unclear. Arguments for ‘ambiguous sign/trend’ are similar; multiple causes of asthma may have different signs and it is unknown which will dominate. One generalist suggested that effects could be different, possibly opposite, in summer and in winter; the “time integration” is therefore uncertain.

Participating experts deemed climate health impacts via hay fever likely through increase in the length of the pollen season and promoted spreading of new, highly allergenic plants (e.g. ambrosia/ragweed, spreading pellitory, olive tree). Indications exist that climate-related factors affect pollen allergenicity and abundance. However, data is sparse and the interplay of relevant factors and magnitude of impacts were seen as unclear. Observed effects differ per plant species and pollen counting station. Furthermore, the effect of longer pollen seasons on the duration and intensity of exposure is unclear, allergy is multi-factorial, and the impacts largely depend on the response of patients, medication use, and the medical sector (e.g. knowledge development and communication).

### Pests

Climate change may affect health-related pests, such as wasps (stings, allergic reactions) and the oak processionary caterpillar (airborne urticating hairs). They scored 2½ (i.q.: 2–3) and 4 (i.q.: 4–4) respectively.

Two respondents, scoring wasps at 2, noted that in recent years, queen wasps woke up earlier in spring after hibernation due to high temperatures in winter and early spring. Combined with good weather conditions during the most vulnerable phase (April), this resulted in increased numbers of wasp nests and wasps. However, frequent warm winters might also reduce winter survival when hibernation is disturbed during a warm episode that is followed by a colder episode. Higher scores were justified by “recent observations”.

The oak processionary caterpillar entered the south of the Netherlands in the 1990s and gradually spread north. Respondents expected a further spread and significant increase in population size due to climate change. Rough disease estimates exist, but the exact potential future magnitude is unknown.

### Vector-borne diseases

Endemic (primarily Lyme disease) and non-endemic vector-borne diseases (e.g. dengue, West-Nile virus, malaria, tick-borne encephalitis (TBE), and leishmaniasis) may be affected by climate change. The survey distinguished between incidents and epidemics; some diseases likely cannot become epidemic for instance because they are easily countered by a well-equipped health care system. Endemic diseases scored 3 (i.q.: 2–4); non-endemic incidents and epidemics scored 3 (i.q.: 2–3) and 2½ (i.q.: 2–3) respectively.

Respondents noted that changes in temperature and relative humidity affect ticks and insects. Lyme incidence has strongly increased in recent years, but many respondents stressed that recent changes were not solely, or even not mainly, caused by climate change. Arguments for ‘ambiguous sign/trend’ (score 2) included the short period of data for the Netherlands, the multifactoriality (e.g. trends in socio-economic factors, land use, contact with vectors, recreation, global travel/trade, welfare, health care), and the unclear effect of climate change on a complex transmission cycle and disease ecology. A subject-matter expert noted that climate change is unlikely to have unidirectional effects on the complex interactions between vectors, reservoirs, humans, and their environments. Arguments for a score of 3 are similar. One subject-matter expert noted ongoing research indicating a longer activity season for ticks in the Netherlands, during warm winters. The subject-matter expert scoring 4 suggested that some data exists and rough estimations could be made.

Arguments for non-endemic diseases are similar. Those scoring 2 argued that many non-climatic factors are likely more important, that the complexity of the diseases makes unidirectional impacts unlikely despite the sensitivity of biological processes to climate. Those who scored 3 acknowledged these difficulties but argued that the risks may increase due to more favourable conditions (particularly for incidental occurrence). One subject-matter expert scored 1–4, noting that the scoring would differ per disease. The arguments seemed to suggest that impacts on some diseases could be considered negligible because other factors presumably dominated disease risks, while for others the effects would be highly uncertain. For epidemics, some respondents shifted to lower scores, adding that this would be dependent on even more variables than incidents.

### Food- and waterborne diseases

Climate change impact on contamination of swimming/recreation water (e.g. cyanobacteria) scored 4 (i.q.: 3–4); other food- and waterborne diseases 3 (i.q.: 3–4).

Regarding food poisoning, arguments for ‘expected sign/trend’ noted a potential effect, but indicated that many other factors (e.g. hygiene codes, refrigeration) determine whether this increases risks. Arguments for ‘order-of-magnitude’ suggest that there is much data on the present relation between temperature and food poisoning, particularly for Salmonella, and that models for impact assessment are available.

For Legionnella, one subject-matter expert, scoring 4, indicated that data and models exist and rough estimates could be made. The majority of generalists, scoring 3, suggested that this effect is related to warm water systems the climate impact on these is unclear, and that this depends on the water distribution systems infrastructure and (autonomous) adaptive capacity.

Regarding contamination of swimming/recreation water, those scoring 4 referred again to the existence of models and data. Those scoring 3 highlighted uncertainties such as the precise nature, extent, and speed of impacts, disease incidence, and changes in the amount of water in urban areas.

### Air quality

Temperature and other weather conditions influence air quality, such as ozone (O_3_) and particulate matter (PM) concentrations. These effects were scored 4 (i.q.: 3–4).

High scores (score ≥4) were justified by known exposure-response relationships of air pollution, and by availability of many data and assessment models. However, estimating the effect of climate change on pollutant concentrations, and speed of changes, was deemed difficult. One subject-matter expert noted that population vulnerability is temperature-dependent and might therefore also change. Lower scores (score 2–3) pointed out that concentrations of ozone precursors might change, countervailing effects exist, and the “time-integrated sign of change” of pollutants was deemed unknown. The latter may refer to summer versus winter effects.

### Flooding and storms

Storms and changes of flooding, due to sea level rise and increased river peak discharges, may have health consequences. Flood-related mortality scored notably wide: 4 (i.q.: 2¼-4⅞). Exposure to contaminants scored 2 (i.q.: 2–3).

For flood-related mortality, arguments for ‘bounds’ (score 5) estimates indicated that many data and models are available, and that we have sufficient experience to estimate this risk. One respondent, scoring 4–5 suggested that scenario-based bounds estimates could be made, but that he would be sceptical about these, because they depend on many assumptions and less quantifiable variables. A respondent scoring 4 estimated that the effects would remain low due to a good evacuation infrastructure and ongoing water-related adaptation. An expert scoring 2 indicated not to know of any “records” on flood-related health impacts of climate change, and that flood-intensity depends on, and is likely dominated by, many non-climatic factors.

Regarding flood-related infectious diseases and exposure to contaminants, respondents scoring 3 noted that some data and models are available. The risk of sewage overflows could increase, thus increasing disease risk. Those scoring 2 stated that knowledge on flood-related infections is mainly from disasters abroad, particularly from developing countries not representative for the Netherlands where the emergency and healthcare system differs.

Flood-related respiratory problems could occur due to moulds in damp homes. Those scoring 3 assessed that it is difficult to translate increased flood risks to additional home dampness and the effects thereof. A subject-matter expert scoring 4 stated that some estimates regarding the current dampness situation do exist.

Studies have shown mental health impacts following floods and evacuations. However, most respondents maintained that the available data is insufficient to make estimations for the future.

Concerning storm-related mortality and injury, most respondents noted that expected changes in storm climate due to climate change are relatively small and highly uncertain, and data is lacking on the effects on mortality and injury. A respondent scoring 5 suggested that data is available and can be extrapolated.

### UV

Climate change may indirectly affect exposure to UV-radiation, for example via changes in cloud cover, ozone-fluxes, and behaviour (e.g. recreational), or due to slowing the recovery of the ozone layer. Respondents were strongly divided over the level of precision.

Two lines of reasoning could be discerned. Arguments for low scores indicated that interactions between climate change and ozone/UV are highly complex, uncertain, and dependent on many other factors. Conversely, arguments for high scores posited that data is available from countries with climate conditions similar to that projected for the Netherlands. Furthermore, good models are available for impact assessment. The main contention seemed to be whether future exposure estimates can be constructed. Some argued that they cannot, while others assessed that they can be extrapolated from present data. Weakening of the immune system scored lower than cataract and skin cancer; one respondent indicated that the effects of UV-radiation on the immune system are uncertain.

### Relevance of health effects for adaptation

**Figure 1:**
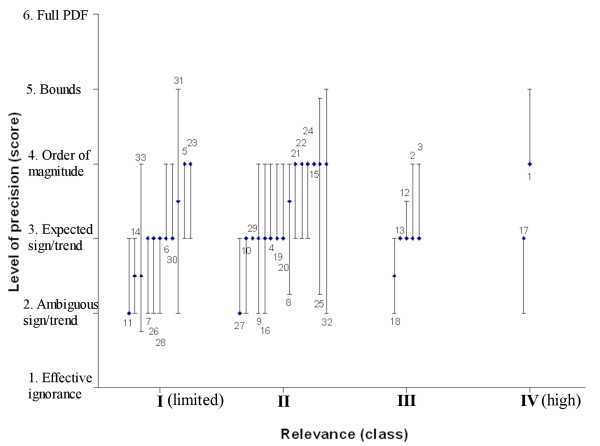
**Level of Precision (points: median scores, error bars: interquartile ranges) of health effects versus their relative relevance, ranging from limited (in no one's top-five) to high (often selected). Numbers 1–33 refer to Table**2.

Heat-related mortality (effect 1) and incidents of non-endemic vector-borne diseases (effect 17) scored highest on relevance. Both were categorised in ‘relevance class’ IV (Figure 1 and table 3). Interestingly, they differ strongly in their level of precision. Other relevant effects (class III) were: non-endemic epidemics (effect 18), heat-related cardiovascular and respiratory problems (effects 2–3) and hay fever (effects 12–13). The arguments for these effects are discussed below (other effects: see Additional file 1).

**Table 3:** Relevance of health effects for Dutch climate adaptation policy

**Effect:**		**Relevance**^**a**^					**Points**^**b**^	**Class**^**c**^
		**1**	**2**	**3**	**4**	**5**		
1	Temperature: Heat-related mortality	6	2	1			41	IV
2	Temperature: Heat-related cardiovascular problems	1		2			11	III
3	Temperature: Heat-related respiratory problems	1	1		1		11	III
4	Temperature: Heat-related stress and sleep disturbance	1					5	II
5	Temperature: Cold-related mortality							I
6	Temperature: Cold-related diseases							I
7	Temperature: Drought-related exposure to contaminants							I
8	Temperature: Shortages of drinking water					1	1	II
9	Temperature: Dehydration		2				8	II
10	Allergies: Asthma			1		1	4	II
11	Allergies: Allergic eczema							I
12	Allergies: Hay fever: duration of pollen season		2		1	2	12	III
13	Allergies: Hay fever: pollen types, abundance and allergenicity		2	1			11	III
14	Pests: Wasps							I
15	Pests: Oak processionary caterpillar		1				4	II
16	Vector-borne: Native vector-borne diseases	1	1			1	10	II
17	Vector-borne: Incidents of non-native vector-borne diseases	1	2	2	1		21	IV
18	Vector-borne: Epidemics of non-native vector-borne diseases	2			2		14	III
19	Food/water-borne: Food poisoning				1		2	II
20	Food/water-borne: Legionnaires Disease					1	1	II
21	Food/water-borne: Contamination of swimming/recreation water				1	2	4	II
22	Air quality: Respiratory problems due to ground-level ozone			1	2		7	II
23	Air quality: Respiratory problems due to particulate matter							I
24	Air quality: Air quality-related cardiovascular problems				1	1	3	II
25	Flood/storm: Flood-related mortality	1		1	1		10	II
26	Flood/storm: Flood-related infectious diseases							I
27	Flood/storm: Flood-related exposure to dangerous substances and contaminants			1			3	II
28	Flood/storm: Flood-related respiratory problems							I
29	Flood/storm: Flood-related mental health problems		1			2	6	II
30	Flood/storm: Storm-related mortality and injury							I
31	UV: Cataract							I
32	UV: Skin cancer			2			6	II
33	UV: Weakening of the immune system							I
34	OTHER: societal disruption elsewhere	1					5	II

^a^The number of times an effect has been selected as 1st, 2nd, etc. most important by the participants. ^b^The point total, where every score of 1st is 5 points, 2nd is 4 points, etc. ^c^The ‘Relevance Class’ resulting from the Points is indicated as: I: 0 points, II: 1–10 points, III: 11–20 points, IV: ≥21 points.

Regarding heat-related mortality, respondents indicated that nursery homes, houses, and urban planning are currently not adapted to high temperatures at all. Other reasons for its relevance include: political interest, public perception, possible stress on the health care system, current lack of interest in this topic in the health care sector, the many people at risk, and the potential for many victims in a short time-period. Regarding heat-related cardiovascular and respiratory problems, participants noted that effects could be substantial, and that many other risk factors could enhance the impact (e.g. traffic, city design, obesity, diabetes).

Regarding non-endemic vector-borne diseases, respondents noted that the impacts could be substantial and difficult to adapt to, and referred to public perception (‘fright factors’ and public unrest). Incidents could be difficult to recognise, and epidemics could place stress on the health system.

Concerning hay fever, respondents pointed to the large number of people affected, considering present-day hay fever incidence. The impact, in terms of health and economic damage (e.g. decreased worker productivity), could be large. For pollen types/abundance/allergenicity, it was noted that the effects could be difficult to adapt to.

## Discussion

### Reflection on findings

Experts’ arguments were generally strong enough to support the interquartile ranges found. Argumentation was more limited for higher/lower scores, for example only referring to “reports” or “opinions”. This makes it difficult to verify the tenability of these scores. The depth of argumentation supporting the 75th percentile score for the heat-related effects (score 5 for mortality, 4 for the other direct effects) seemed relatively limited, referring to literature and experiences with recent heat waves. For flood-related mortality and respiratory problems, the lower scores (score 2–3 and 2 respectively) received limited argumentation.

Recent Dutch impact assessments provide mostly qualitative information on potential effects of local climate change on health; quantitative information relates to the current and historic state of affairs regarding various health issues (e.g. trends in hay fever prevalence). Data seems most advanced for temperature-related mortality, for which scenario-projections exist. Huynen [25] calls these “order-of-magnitude estimates”, which corresponds with our results. For other high-scoring effects, no quantitative estimates have been found. At the international level, McMichael et al. [6] do provide projections for malnutrition, diarrhoea, malaria, floods/landslides (mortality), and temperature-related mortality. Significant caveats are presented for all. IPCC [2] additionally presents the results of modelling studies on other vector-borne diseases (dengue, Lyme, tick-borne encephalitis) and on air quality. For flooding, temperature, and air quality, the level of precision in the literature correspond with the results of this study: rough (‘order of magnitude’) quantitative estimates are possible, but involve considerable caveats. Malnutrition was not included in our study. The studies on vector-borne diseases mostly assess climate suitability and population-at-risk. This seems insufficient to assess the health risks for the Netherlands quantitatively, but such studies can be used to discern whether there may be reason for concern regarding these diseases (and potentially the seriousness under various scenarios, albeit not in terms of a quantitative health risk). This is in agreement with the analyses made by the participants in this study. Menne and Ebi [26] include a temperature-Salmonellosis relation and season-Campylobacteriosis time-series for the Netherlands. Our participants mentioned these relations, but disagreed with each other on whether they can be used straightforwardly for climate impact assessment, considering the many other factors at play.

The ‘Level of Precision’ question was relatively broad. Potentially, some participants could have scored effects assuming standard climate projections (e.g. the Dutch KNMI or global IPCC scenarios), while others could have taken broader ignorance regarding local climatic changes into account. Because the argumentation focused almost exclusively on uncertainties in assessing health impacts (i.e. translating a climatic change into its health impacts), rather than climatic uncertainties, we interpreted the scores as ‘given a climate scenario’. Another consideration in interpreting the results is whether there may have been differences in whether respondents in their scoring have assumed inclusion of non-climatic factors, such as (trends in) the state of the healthcare system, regulations (e.g. on food hygiene), and autonomous adaptation. These can complicate health impact assessments considerably. They are very relevant for assessing the adaptation challenge, but are less indicative of the quality of the knowledge base. The argumentations for the scores provided by the respondents allowed us to explore to what degree such considerations have played a role. We found that such factors appeared most strongly in the argumentations regarding heat (mortality and indirect effects), vector-borne diseases, food- and waterborne diseases, and flooding (mortality). Considering participants’ argumentation, if non-climatic factors were to be explicitly excluded, the lower bounds of the interquartile ranges could be higher for indirect heat-effects and vector-, food- and waterborne diseases. For heat-related mortality this is unlikely considering the body of other arguments. For flood-related mortality it is unclear, due to the fact that respondents provided limited argumentation for lower scores.

One reviewer raised the issue that score ‘order of magnitude’ may have been interpreted by some respondents literally as ‘within a factor 10’ rather than the description given in table 1, implying that higher levels of precision should be reserved for health impacts known within less than a factor 10. Consequently, if very wide-ranging estimates, spanning several orders of magnitude (such as in e.g. imprecise probability assessments), might have been possible for an effect, some respondents may have scored it ‘sign/trend’ rather than ‘order of magnitude’. The original description of ‘first order estimates/order of magnitude’, which was linked to from the questionnaire as background material (see Additional file 1) provided examples in terms of ‘factor of 2’ and ‘power of 10’. However, the definitions shown to respondents (table 2) each time they were asked to score effects, provided a broader definition, including low-precision techniques such as scoring on ordinal scales and comparative qualitative analyses. As such, we expect the effect on the results of this study to remain limited. A related point is whether the order of the scale could affect the scoring by participants. The scale used in this study listed low precision (ignorance) at the top and high (full PDF) at the bottom, whereas the original paper listed high to low. Such order effects could result in slight shifts in the scoring, but we expect the effect on this study to be minor because the scoring was performed by experts in their field and was accompanied by explicit argumentation. Nonetheless, this issue could be relevant for non-argumentative opinion polls, and it would be interesting to study the extent to which order effects apply to this type of scale.Score ‘ambiguous sign/trend’ was often interpreted as ‘unclear whether any impact will take place’, rather than ‘can be positive or negative’. This occurred often when effects were deemed multi-factorial or affected by confounders, or when effects in a wealthy society with well-prepared health and emergency-response systems were deemed unclear. Notable examples include: indirect heat-related effects (e.g. exposure to contaminants), asthma, allergic eczema, and indirect effects of flooding (e.g. infectious diseases). This implies a different level of uncertainty than cases where effects were deemed ‘plausible, but unknown and likely not unidirectional’. Vector-borne diseases and wasps are examples of the latter.

As respondents were asked to what extent they were able to estimate the risk, it is relevant to explore whether the score resulted from the state of knowledge or from the respondent’s personal level of knowledge, skills and familiarity with risk assessment techniques such as modelling, statistical techniques, and expert elicitation. Personal lack of knowledge or skill was explicitly checked in the argumentation as potential bias. It appeared to play a minor role, with lack of knowledge appearing only on a few occasions for scores of 2 or 1. Another measure to the same end was to track scores by generalists and by subject-matter experts separately. These scores corresponded fairly well. Weighting resulted in minor changes (¼-¾) of interquartiles. Medians were affected in a few cases: +½ for flood-related mortality, air quality-related, and UV-related effects. Regarding air quality and flood-related mortality, subject-matter experts scored notably higher than generalists. The awareness of statistical and expert elicitation techniques from outside the disciplines involved in the field of ‘climate change & health’ cannot be determined. However, most of the argumentation focused on the availability of basic data and models, the degree to which the system dynamics are understood, and the knowledge gaps and complexities that exist. As such, the scores should be interpreted as whether it is appropriate to quantify the health risks for specific effects given the state of knowledge, rather than whether it is possible to produce a number in one way or another. In a few instances, for low scoring effects, respondents made arguments that the impacts could be low or high considering e.g. constraints posed by the high quality healthcare system or considering the current incidence. Consequently, it may be possible to further scope some low scoring risks, at least to some extent, using for instance imprecise, ordinal or qualitative/comparative approaches. Further investigation would be required to assess the scope to which this is possible and appropriate.

Scores and arguments for the relevance of effects varied between experts, although the general ordering and, for the high-scoring effects, the general line of reasoning is relatively clear. Results should be seen as indicative, as they may vary over time, group of respondents, and country. An interesting issue, for example, is the potential influence of recent (extreme) events. Such events may influence public perception and therefore the societal salience of effects. Current public perception played a role (although not a major role) in the arguments for heat-related effects, referring to the 2003 European heat wave. It also played a role for vector-borne diseases, although the arguments related to the potential role it could play due to e.g. the ‘fright factors’ associated with the effect, rather than current public perception due to recent events. Recent events might also influence expert scorings when they reveal vulnerabilities that had been unknown or not sufficiently perceived before. Again, this seems to play a role for heat-related effects in reference to the 2003 heat wave. This certainly is a valid reason to consider the effect relevant, and one that may remain relevant over time. However, it does present the interesting question whether such unknown vulnerabilities are (or could be) present for other effects as well. This question is however beyond the scope of the present study.

Being based on expert elicitation, results should be treated with some care. The sample of participants is always a limited subset of the total expert-population and situational factors influence the composition of the panel (e.g., who is well-known in the field, who has time to participate). Therefore, results are not necessarily representative. Rather, they give an approximation, and the lines of reasoning behind the scores provide valuable insights into the issue studied. Given the broad coverage of relevant subfields, relative consistency in scores and arguments for most health effects, and consistency with the literature, we consider the findings robust enough to support the general conclusions.

### Relevance for other countries

Many arguments put forth by participants apply to the wider European and global context, particularly when relating to knowledge gaps and complex multi-factorial relations. The level of precision may differ slightly between countries. Respondents noted in several instances that data was available for other countries, but not for the Netherlands. Specific topics may have been studied in some countries, but not in others: e.g. uncommon events (floods, epidemics), and health effects that are currently particularly important in some countries/regions, but not in others. Similarly, respondents noted that e.g. indirect effects of temperature and flooding were less predictable due to highly developed health care and emergency-response systems. In countries where these systems are weaker, data is available from present-day impacts, resulting to higher levels of precision. Conversely, however, for effects for which effective short-term abatement options exist (e.g. shortages of drinking water), such well-developed systems and available resources could constrain impact-estimates. The geographical level of analysis may also be a relevant factor for determining whether quantification is possible.

### Policy implications

Different adaptation approaches (see Additional file 1: Table S1) are suitable under different levels of uncertainty, such as statistical uncertainty, scenario uncertainty, and recognized ignorance and surprise. This framework, proposed by Dessai and Van der Sluijs [5], links the type of uncertainty that characterises the available knowledge to the suitability of various adaptation approaches (in terms of their capacity to cope with the uncertainties). If statistical uncertainty dominates, well-coping adaptation approaches focus on classic quantitative risk analysis, optimization, and ‘safety margins’. Approaches focusing on dimensioning adaptation measures using scenario-analysis or on exploring the robustness of policy strategies under uncertainty cope well with scenario uncertainty. Under ignorance and surprise, well-coping approaches focus on enhancing society’s (or a policy strategy’s) capacity to tolerate disturbances, to cope with changes and surprise, and to adapt and be adapted.

This heuristic can be loosely connected to the scoring system in table 1, where ignorance takes a more pronounced role in the health risk assessment towards the bottom of the scale and statistical and scenario uncertainties are most pronounced in the top and middle parts. It should however be noted that all three types of uncertainty are usually present and may hold policy-relevance in one way or another. For instance, even under fully quantifiable risks with statistical uncertainties as most pronounced, remaining ignorance may give rise to a ‘surprise scenario’ that is relevant enough to keep in mind. Conversely, even if knowledge gaps and ignorance make it impossible to quantify health risks, it may still be possible to make quantitative explorations of other metrics. For instance, while quantitative assessment of health risks for vector-borne diseases seems unfeasible, scenario studies have been performed on the population potentially at risk to diseases, e.g. due to changing climatic suitability of various countries for the diseases and their vectors. Such a focus on vulnerability, rather than health risk, seems to be able to circumvent some barriers to quantification, and provides useful information that could contribute to some tailoring and prioritization within any adaptation approach. It seems useful to further investigate the options to analyse the relative vulnerability of populations, specific regions, policy proposals, and societal developments (e.g. for healthcare policies or urban development) to various health-related climate change effects. Another point worth mentioning is that the level of precision of health risk estimates, as reported in this paper, may change over time, due to progressing knowledge on both the health effects and their uncertainties. Regarding the latter, this paper presents a first broad analysis and comparison, but further in-depth studies on the separate effects will be required. It would be interesting to examine the available evidence in more detailed way and explore what metrics (health risk, vulnerability, etc.) can be meaningfully assessed and which analytical approaches are appropriate. Further analyses might include expert elicitations, modelling, statistical techniques, or a combination of these; the expert elicitations might involve quantitative approaches (eliciting e.g. PDF, bounds, or order of magnitude), or use semi-quantitative, ordinal or qualitative approaches; e.g. applying scales such as the IPCC’s confidence or likelihood scales (e.g. [27,28]) or fuzzy techniques (e.g. [29]). More detailed exploration of the types of uncertainties that play a role (cf. [5]) would be useful as well. Considering these issues, combinations of policy approaches are worth considering, and it is advisable to incorporate the ability to take onboard progressing insights into policy strategies and their practical implementation. In terms of the overall approach, however, traditional computative optimization approaches to adapting to climate risks under the ‘predict & prevent’ paradigm are particularly suitable for levels of precision of ‘bounds’ to ‘full PDF’ (score 5–6), but perform poorly under deep scientific uncertainty and knowledge gaps. For ‘order-of-magnitude’ to ‘bounds’ impacts (score 4–5), robust decision-making is often a suitable approach. For ‘order-of-magnitude’ and lower (score 1–4), enhancing resilience, flexibility, and adaptive capacity^c^ are recommendable approaches.

As one reviewer pointed out, the precision of the estimate is not the only factor to be considered when choosing an adequate adaptation strategy. It needs to be considered together with the closeness of the estimated magnitude to a level of concern, as together they indicate the likelihood that a level of concern might be exceeded. Other factors also deserve consideration, for example the severity of the health effect or the risks of overinvestment. For instance, the implementation of resilience based strategies for health effects with low precision ratings will still require quantitative decisions to be made (e.g. how much over-capacity to provide in the health system), which will inevitably require some judgement about the upper bound of health effects, taking due account of its imprecision.

Some potential approaches focus on making specific adaptations to particular impacts, while others deal with general capacity building and options that effect a range of health issues. For resilience, a distinction can also be made between specified resilience, of particular parts of a system to specific disturbances, and general resilience [30]. Specific adaptation options often focus on the near-term and local scale, while general resilience incorporates broader considerations, including other geographical and temporal scales, pressures other than climate change, and novel shocks [30,31]. Consequently, options that increase general resilience and capacity building are useful under ignorance and surprise: facilitating adaptation when impacts are greater or different than expected and providing some level of no-regret, by contributing to system-health in general, when specific impacts turn out to be limited in retrospect. Specific adaptations may run the risk of proving an overinvestment (financially as well as in terms of other efforts) when impacts remain limited and generally offer no protection against unanticipated changes. In the questionnaire, some experts warned against overly-specific measures and (difficult to modify) ‘hard-engineering’ options as vulnerable to surprise. Nonetheless, it is difficult to assess and indicate the extent to which general capacity building reduces specific climate risks. Therefore, more specific measures are useful for impacts that are more readily quantifiable or are relatively policy-relevant. Considerations such as costs, potential side-effects, encroachment on society, and extensiveness of interventions (socio-economic, structural and political efforts/impacts) are also important. Costly or extensive/far-reaching options are worth considering, from a decision-maker’s point of view, when the impact is considered highly relevant. It is however important to critically reflect on one’s goals and the extent to which the options contribute to these goals. For instance, if an effect is relevant primarily due to public perception rather than the health impact, one might wonder whether highly costly options are prudent; would the money not be better spent on issues that have higher health benefits? This is largely a political choice, but it is important to make this choice explicitly. The specific reasons for relevance can offer some clues as to what options might be useful. High public concern due to recent events might evaporate over time or could quickly change when other concerns, such as an economic crisis, take precedence. In other words, the level of concern might be uncertain in the long run. Consequently, one might opt for options that improve the health system in general, enhance its flexibility, or have co-benefits. If (potential) concern is due to ‘fright factors’, such as respondents suggested for vector-borne diseases, this concern might be more robust over time, as fright factors involve basic human psychology. In such cases, investing in public communication mechanisms and plans for use during outbreaks might be useful. Concern (e.g. expert concern; in this study noted in relation to the 2003 European Heat Wave) due to recent events revealing unknown vulnerabilities, and the notion of current unpreparedness, might prompt not only measures to reduce the impact of this effect, but also further research into why these vulnerabilities arise. They might also prompt research into vulnerabilities for other effects; if vulnerabilities turn out higher or different for one effect, they might also do so for other effects. In addition to the various points above, some options could be considered no-regret, even if very specific, if they also address existing climate risks or provide co-benefits in other policy fields. For instance, local adaptive efforts are envisaged in Dutch and other European cities even in cases where the sense of urgency is low; such measures often focus on other policy goals, such as biodiversity conservation or improving quality of public spaces, with adaptation as co-benefit [32,33]. Such options can make economic or societal sense irrespective of future climate change. These points are summarised in table 4.

**Table 4:** Implications of uncertainty and relevance for policy

***Effects are of:***	**Low relevance**	**High relevance**
**High level of precision**	Tailored, prediction-based strategies (e.g. risk approach) are feasible.	Tailored, prediction-based strategies (e.g. risk approach) are feasible.
	Focus: low costs/efforts or co-benefits.	Consider (but critically reflect on) costly and extensive options.
**Low level of precision**	Enhance system’s capability of dealing with changes, uncertainties, and surprises (e.g. resilience approach).	Enhance system’s capability of dealing with changes, uncertainties, and surprises (e.g. resilience approach).
	Focus: low costs/efforts or co-benefits.	Consider (but critically reflect on) costly and extensive options, including precautionary measures. Assess overinvestment risks and flexibility.

Considering the above, strategies that enhance resilience, flexibility, and adaptive capacity seem most appropriate for the majority of health effects. For effects that are highly policy-relevant, such as non-endemic vector-borne diseases, precautionary and other rigorous/costly options could also be considered. However, for such options, it would be advisable to assess the risks of overinvestment and improve their flexibility. For many health effects, climate change worsens already existing effects; some options would be beneficial anyway, regardless of climate change. We advise assessing the availability of ‘no-regret’ options and the ‘climate and health’ co-benefits of policy on other policy-issues. For quantifiable health effects, such as heat-related mortality, it seems useful to combine system-enhancement with approaches such as ‘robust decision-making’, which entails exploring the ability of adaptation packages, or the current health system or society, to function under a range of plausible futures. Knowledge gaps on the effectiveness of adaptation options will likely limit this to a qualitative/semi-quantitative exploration at present. Such an exploration on uncertainty typology could contribute to policy/political discussions on the preferred ambition level of adaptation strategies, also considering the range of potential impacts.

## Conclusions

Knowledge regarding health risks of climate change is characterised by large gaps and deep uncertainties. Planned adaptation to these risks requires profound understanding of the level of uncertainty of available knowledge of anticipated health effects. This study presents a systematic exploration and appraisal of uncertainties regarding climate change-related health risks. Using a six point scale, experts were asked to indicate the level of precision with which health risk estimates can be made, given the present state of knowledge. The study focussed on The Netherlands.

The experts assessed that, for most of the 33 (potential) health effects identified, it is possible to indicate its sign of change, but not its magnitude. Individual scores varied, generally between being unable to indicate the direction of change and being able to calculate the rough ‘order-of-magnitude’ of the impacts. Factors that were often indicated to limit quantification include: limited data (in general and country-specific), the multi-factorial nature of the health issues (many important non-climatic drivers of change), and unknown impacts considering a high-quality health system.

For some effects, rough estimates of the order-of-magnitude were deemed possible: heat- and cold-related mortality, the oak processionary caterpillar, microbial contamination of swimming/recreation water, flood-related mortality and air quality-related effects. For these effects, data and impact assessment models are available. However, the availability of locally-specific data is relatively limited, there are many confounding factors, present-day response-relationships may change, and changes in local extreme weather events, such as heat waves, are still difficult to project for the future.

For allergic eczema, flood-related exposure to dangerous substances, wasps, UV-related weakening of the immune system, and epidemics of non-endemic vector-borne diseases it may not be possible to even indicate the direction of change. The latter, however, differs per specific disease: for some, effects are unlikely, for others, unknown. In addition to the difficulties noted above, the cause-effect relations of these effects are often highly complex and impacts are likely multi-directional.

These results suggest that, among various alternative approaches to climate change adaptation under uncertainty, approaches that focus on enhancing the health system’s and society’s capability of dealing with changes, uncertainties and surprises (for example by increasing resilience, flexibility, and adaptive capacity) are most suitable for adapting to the health impacts of climate change. Furthermore, we advise assessing the availability of ‘no-regret’ options, which make economic or societal sense due to co-benefits or health benefits in the current climate, and the ‘climate and health’ co-benefits of adaptation policy on other policy-issues. For more quantifiable effects, we recommend exploring the robustness of various policy strategies under a range of plausible outcomes, at least in a qualitative/semi-quantitative way. Such analyses can contribute to setting preferred levels of ambition for adaptation efforts. For highly relevant effects, precautionary measures and other highly specific, costly or rigorous adaptations are also a relevant option, although it is advisable to enhance the flexibility of such options and to assess the associated risks (e.g. of these options becoming an overinvestment or resulting in detrimental side-effects).

Because nature, extent and rate of climate change and its health impacts are uncertain, understanding the relative level of relevance and uncertainty is crucial to making rational choices in adaptation policies and for possible adjustments if climate change effects occur slower, faster, or just different than earlier expected. Similar to e.g. Ebi [34] we argue that, to reduce climate change-related health risks, flexible, adaptive, multilevel and dynamic adaptation strategies should be developed. This study indicated that analysing and characterising uncertainty by means of a typology can be a very useful approach for selection and prioritization of preferred adaptation policies to reduce future climate related health risks.

## Endnotes

a. Statistical uncertainty implies being able to specify an outcome (e.g. a disease estimate) as well as its probability (e.g. 95 % confidence interval). Scenario uncertainty implies being able to specify multiple alternative outcomes but not their relative probability. Recognized ignorance & surprise imply that both outcomes and probabilities are unclear (e.g. not quantifiable, hypothetical, or unknown).

b. The literal formulation of the ‘relevance to adaptation’ question was: “In the following questions, you will be asked to zoom in on the top five most relevant health effects (of climate change) for climate change adaptation in the Netherlands in view of public health and to examine the uncertainties more closely. In estimating what health effects are most ‘relevant’ for Dutch climate change adaptation, take into account the possible magnitude of the health impact, economic impact, public and political perception, and the availability of options for adaptation and control.”

c. Resilience: the ability of a system to tolerate disturbance without collapsing into a qualitatively different state; to withstand shocks and rebuild itself when necessary. In social systems, it also involves the capacity to anticipate and plan for the future. Entails e.g. quick responses, fast recovery following shocks, enhanced coping capacity through e.g. buffers or redundancy; limiting the impacts of health effects. Adaptive capacity: society’s ability to adapt to changes. Often relates to the availability of resources (e.g. funds, social capital, institutional capacity, knowledge). Flexibility: whether an option/strategy can be easily modified should this be required in the future, or enhances the flexibility of the health care system itself.

## Abbreviations

IPCC, Intergovernmental Panel on Climate Change; i.q., Interquartile range; KNMI, Royal Netherlands Meteorological Institute; PDF, Probability density function; PM, Particulate matter; O_3_, Ozone; UV, Ultraviolet (radiation).

## Competing interests

The authors declare that they have no competing interests.

## Author contributions

AW, AdJ, LvB, and JvdS designed the research. AW performed the research and analysed the data. AW, AdJ, LvB, WT, and JvdS wrote the paper. All authors read and approved the final manuscript.

## Supplementary Material

Additional file 1 Supplementary Material for paper: “Health risks of climate change: An assessment of uncertainties and its implications for adaptation policies.” Click here for file
