# Supplementary material for: Health risks of climate change: An assessment of uncertainties and its implications for adaptation policies
Source: Environ Health. 2012 Sep 19;11:67. doi: 10.1186/1476-069X-11-67 (PMC3506559; doi:10.1186/1476-069X-11-67)
Supplement: Additional file 1 — Supplementary Material for paper: “Health risks of climate change: An assessment of uncertainties and its implications for adaptation policies.” [file 1476-069X-11-67-S1.pdf]

Supplementary Material for paper: **"Health risks of climate change: An assessment of uncertainties and its implications for adaptation policies"**

Supplementary Methods

Table S1. Qualitative indication of how well various adaptation approaches can deal with three levels of uncertainty, according to Dessai and Van der Sluijs (2007). Legend: ++ very good, + good, ± somewhat, - bad, -- very bad.

Frameworks for decision making under uncertainty	Statistical uncertainty	Scenario uncertainty	Recognized ignorance & surprises
Top-down approaches (predictive):			
IPCC approach	+	++	--
Risk approaches	++	+	--
Bottom-up approaches (resilience-oriented):			
Engineering safety margin	++	±	-
Anticipating design	++	+	+
Resilience	±	+	++
Adaptive management[1]	++	-	--
Prevention Principle	++	±	--
Precautionary principle	+	++	++
Human development approaches	±	+	+
Mixed approaches:			
Adaptation Policy Framework	+	+	+
Robust decision-making	+	++	+

[1] There are two distinct interpretations of adaptive management: (a) experimental policy interventions aimed at increasing knowledge of the managed system, and (b) a holistic, flexible and continually updated approach to policymaking. This table refers to interpretation (a).

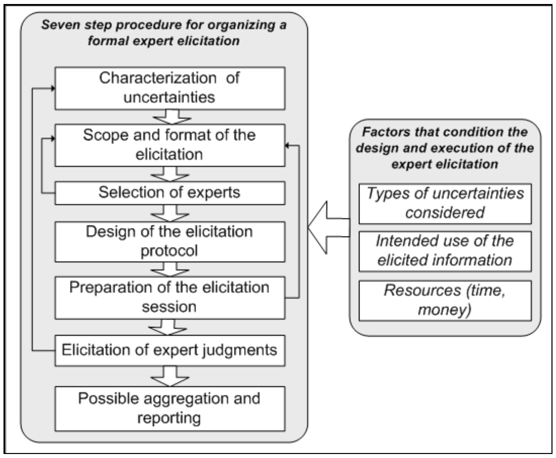

Figure S1. Steps in an expert elicitation. Source: Knol et al. (2010), Environ. Health 9:19.

List of participants

- Neil Adger (University of East Anglia, UK)
- Inez de Boer (The Netherlands Red Cross)
- Ides Boone (Veterinary and Agrochemical Research Centre, Belgium)
- Marieta Braks (National Institute for Public Health and the Environment (RIVM))
- Leendert van Bree (Netherlands Environmental Assessment Agency)
- Bram Bregman (Royal Netherlands Meteorological Institute (KNMI))
- Bert Brunekreef (Institute for Risk Assessment Sciences, Utrecht University)
- Hein Daanen (TNO; e-mail response only)
- Guus de Hollander (Netherlands Environmental Assessment Agency)
- Guy Hendrickx (Avia-Gis, Belgium)
- Paul Heyman (Queen Astrid Military Hospital, Belgium)
- Maud Huynen (International Centre for Integrated Assessment & Sustainable Development, Maastricht University)
- Fokke de Jong (Climate changes Spatial Planning; Alterra)
- Loïc Josseran (Institut de Veille Sanitaire, France)
- W.F. Passchier (Department of Health Risk Analysis & Toxicology, Maastricht University)
- J. Schols (Department of General Practice, Maastricht University)
- Aad Sedee (Alterra)
- Tom van Teunenbroek (Ministry of Housing, Spatial Planning and the Environment)
- Arnold van Vliet (Environmental Systems Analysis Group, Wageningen University)
- Arjan Wardekker (Copernicus Institute for Sustainable Development and Innovation, Utrecht University)
- Letty de Weger (Leiden University Medical Center)

Questionnaire

Note that this is a reduced version of the full survey; repeating questions are not shown, but are indicated.

I. Introduction

1. What is your name? (for identification and acknowledgement; results will be anonymised)

2. In case you've received the link to this survey via a colleague rather than an e-mail from the research team, please indicate your e-mail address.

3. What would you consider to be your expertise regarding climate change and health? [mark all that apply with 'x']

	Generalist or expert on climate (change) adaptation
	Expert on health and climate (change) adaptation
	Generalist knowledge on climate (change) and health, or one or more topics in this field.
	Expert on temperature-related health effects
	Expert on allergies
	Expert on pests (wasps, oak processionary caterpillar)
	Expert on vector-borne diseases
	Expert on food- and water-borne diseases
	Expert on air quality-related health effects
	Expert on health effects due to flooding and storm
	Expert on UV-related health effects
	Other:

4. What is your professional background? [mark all that apply with 'x']

	Scientist
	Policymaker
	Policy advisor
	Health practitioner (medical professional, GGD/public health services, etc.)
	Other:

5. This study will focus on the Netherlands. As the number of Dutch experts on the topic of 'climate change & health' is limited, we've also invited experts from other countries. Please indicate your background. [mark one that applies with 'x']

	Dutch, and have specific expertise or experience on this topic in the Netherlands
	Dutch, no specific expertise or experience on this topic in the Netherlands
	Non-Dutch, but have specific expertise or experience on this topic in the Netherlands
	Non-Dutch, no specific expertise or experience on this topic in the Netherlands

II. Level of Precision of health risk estimates

In the following sections (per category of effects), you will be asked to indicate the level of precision with which you could estimate the magnitude of each health risk for a number of specific health issues (also take into account interactions between issues), at the present state of knowledge. Assume you would be given some time to review the relevant literature, before you would make the effect estimate. The level of precision will be rated on a scale based on Risbey & Kandlikar (Climatic Change, 2007). A brief description will be provided on each of the following pages. A full description can be found at: [*link included in original survey*]

This section is divided into nine specific subtopics: a. temperature, b. allergies, c. pests, d. vector-borne diseases, e. food/water-borne diseases, f. air quality-related, g. flooding/storm, h. UV-related, i. (other).

IIa. Temperature-related health effects

In this section, you will be asked to indicate the level of precision for health risk estimates regarding climate change & temperature.

**Rating:**	**Label:**	**Description:**
1	Effective ignorance	Knowledge of the factors that govern this effect is so weak that we are effectively ignorant.
2	Ambiguous sign or trend	Some effect is expected, but its sign or trend is not clear. There are plausible arguments either direction (effect could be positive, could be negative; could increase or decrease).
3	Expected sign or trend	It is clear what the sign and trend of the effect will be. However, there is no plausible or reliable information on how strong it will be.
4	Order of magnitude	It is possible to give a rough indication of the magnitude of the effect, a qualitative scoring (e.g. 1-10 scale), or a rough comparison with other effects.
5	Bounds	It is possible to estimate the bounds for the distribution of the effect, e.g. its 5/95 percentiles (effect is only 5% likely to be more than … and only 5% likely to be less than …). However, the shape of the distribution, or best-guess estimates, cannot be provided.
6	Full probability density function	It is possible to provide a full probability density function; the bounds as well as the shape of the distribution.
N/A	Don't know / no answer	

6. Regarding the following specific health issues, with what level of precision would you be able to estimate the magnitude of the health risk for the Netherlands (due to climate change)? Assume you would be given some time to review the relevant literature, before you would make the effect estimate. Use the scale above. [per health issue, mark your rating with 'x']

	1	2	3	4	5	6	N/A
Heat-related mortality							
Heat-related cardiovascular problems (Dutch: hart- en vaatziekten)							
Heat-related respiratory problems							
Heat-related stress and sleep disturbance							
Cold-related mortality (decrease)							
Cold-related diseases (e.g. influenza) (decrease)							
Drought-related exposure to contaminants (less dilution of pollutants during extreme droughts)							
Shortage of drinking water							
Dehydration							

Please provide a brief argumentation for your rating above (if any), and if possible, provide some literature references in support.

7. Argumentation and references for 'heat-related mortality':

[REPEAT Q7 FOR ALL OTHER HEALTH EFFECTS UNDER 'TEMPERATURE']

[REPEAT ABOVE FOR ALL OTHER HEALTH THEMES:]

IIg. Other

47. Are there any other important health issues for the Netherlands (due to climate change) that were not included in the questions above? If so, please indicate these effects plus their level of precision for health risk estimates.

III. Key uncertainties

In the following questions, you will be asked to zoom in on the top five most relevant health effects (of climate change) for climate change adaptation in the Netherlands in view of public health and to examine the uncertainties more closely.
In estimating what health effects are most 'relevant' for Dutch climate change adaptation, take into account the possible magnitude of the health impact, economic impact, public and political perception, and the availability of options for adaptation and control.

Shortlist of health issues [*see main text of paper, as well as Table S2*]

48. Most relevant effect: [indicate the number from the list above]

49. What makes this effect relevant for the Netherlands (brief description or keywords suffices)?

50. Please describe the key uncertainties that play a role in estimating the magnitude of this health risk. If possible, indicate relevant literature references.

51. Could you describe which adaptation options/strategies would be particularly well-capable of dealing with these uncertainties and which would be very vulnerable to them (and why?)?

[REPEAT ABOVE FOR 2ND, 3RD, 4TH, AND 5TH MOST RELEVANT HEALTH EFFECTS]

If there is anything else you would like to add, suggest or clarify regarding climate change, health, adaptation and uncertainties, you can do so in the field below.

68. Any other things you would like to add, suggest or clarify?

[END OF SURVEY]

Briefing note attached to invitation and questionnaire

Briefing note for expert-survey 'climate change, uncertainties and health'

It is increasingly recognized that adaptation to climate change has become unavoidable. It is the only response available for the impacts that will occur over the next several decades before mitigation measures can have an effect. Anticipatory adaptation however is made difficult by substantial uncertainties, both quantifiable and unquantifiable. These arise in many levels of climate impact assessment and adaptation policy (e.g., uncertainties in future emissions, response of the climate system, regional and local effects, local vulnerability, and effectiveness of adaptation options). These add up to what has been described as a 'cascade' or 'explosion' of uncertainties.

Thus, important questions for climate change adaptation include: what is the nature and level of uncertainties for specific themes (water management, health, etc.), and how can climate change adaptation cope with these uncertainties?

Project background

The Netherlands Environmental Assessment Agency commissioned the Copernicus Institute at Utrecht University to examine this issue. In 2007, a theoretical 'Scoping Study' was prepared, as a joint project with the Tyndall Institute and the University of East Anglia (Norwich, UK).

As a follow-up, a series of practical case-studies for the Netherlands is being performed. Cases include: (1) *water safety and management*: a critical evaluation of the 2008 Delta Committee advice based on the strategies suggested in the Scoping Study, (2) *nature*: an in-depth examination of resilience as a strategy for the Wadden Sea region, and (3) *public health*: the present expert-survey on the uncertainties involved and options for uncertainty-robust adaptation strategies.

The results of these studies will be used as input for (strategic and specific) climate policy analyses and advice produced by the Netherlands Environmental Assessment Agency for the national government of the Netherlands. The results of the case-studies will be published as a scientific report (in Dutch). We also aim to write several articles for international peer-reviewed scientific journals, based on the cases.

Survey setup

The survey aims to examine the uncertainties related to 'climate change & health' in the Netherlands, the possible relevance of these uncertainties for Dutch climate change adaptation policy, and into uncertainty-robust adaptation strategies. Both Dutch and international scientists and professionals are invited to participate. The survey will focus on two issues:

- Possible **level of precision** for health risk estimates. The best-fitting adaptation strategy depends on the level of uncertainty. This section is intended as a broad scanning of the entire field of climate change & health. It examines the level of uncertainty for various categories of effects. Specific health risks are assessed using a 'level of precision' scale based on Risbey & Kandlikar (2007) plus argumentation.
- Most **relevant uncertainties** and **uncertainty-robust adaptation strategies**. This section will ask you to zoom in on the top-5 most relevant health risks for climate change adaptation in the Netherlands. You will be asked to further specify the uncertainties for these, and to describe which adaptation strategies are robust to the uncertainties, and which are vulnerable.

Background documents

Dessai, S., and J.P. van der Sluijs (2007). "Uncertainty and Climate Change Adaptation: a Scoping Study". http://www.nusap.net/downloads/reports/ucca_scoping_study.pdf

Risbey, J.S., and M. Kandlikar (2007). "Expressions of likelihood and confidence in the IPCC uncertainty assessment process". Climatic Change, vol. 85, pp. 19-31.

Survey addresses:

Online: http://www.copernicus.uu.nl/phpESP/public/survey.php?name=ClimateUncertaintyHealth

Word version: http://www.copernicus.uu.nl/climaterisk/SurveyClimateUncertaintyHealth.doc

Full description of 'level of precision' scale as attached to the questionnaire

Level	Measure of likelihood	Justification	Explanation of step
-	-	-	First define the variable or outcome to be examined and the context in which it is being examined, in order to:<br>• ensure that the outcome in question has a commonly shared understanding and can be meaningfully quantified;<br>• facilitate comparison of uncertainties across studies and through time
1	Full probability density function	Robust, well-defended distribution	This is the full likelihood description. This serves to capture either those variables for which historical data exists, or those for which there is sufficient consensus.<br>• Is it reasonable to specify a full probability distribution for the outcome? If yes, specify the distribution. Justify your choice of distribution and 5/95 percentiles.<br>• Are there any processes or assumptions that would cause the 5/95 percentiles to be much wider than you have stated?<br>• If you cannot provide justifications for why you consider the distribution shape and 5/95 percentiles to be fairly robust, then move to level 2.
2	Bounds	Well-defended percentile bounds	• Is it reasonable to specify bounds for the distribution of the outcome? If yes, specify 5/95 percentiles.<br>• Can you describe any processes or assumptions that could lead to broader/narrower bounds? If so, describe and revise.<br>• If the bounds are robust to assumptions, then specify your 5/95 bounds and your reasoning for placing them where you did. If you cannot provide bounds confidently then go to level 3.<br>• The choice of 5/95 percentiles is by convention. Other ranges (e.g. 10/90) could also be used by different research communities as long as the choice is clear.
3	First order estimates	Order of magnitude assessment	• If appropriate, specify and justify your choice of a first order estimate for the value of the variable, indicating the main assumptions behind the value given. In specifying a value, do not report more precision than is justified.<br>• For example, if the value is only known to a factor of two or an order of magnitude, then report it in those terms. In some cases, powers of ten may be appropriate; in other cases more nuanced scales may be used so long as they are declared and supported.<br>• How robust is your estimate to underlying assumptions? If it is not particularly robust to the set of assumptions or outcomes you listed, then go to level 4.
4	Expected sign or trend	Well-defended trend expectation	• Can you provide a reasonable estimate of the sign or likely trend (increase, decrease, no change) of the expected change?<br>• If so, give the expected trend and explain the reasoning underlying that expectation and why changes of the opposite sign or trend would generally not be expected.<br>• Describe also any conditions that could lead to a change in trend contrary to expectations.<br>• It is reasonable to include in this category changes which have a fair degree of expectation, but which are not certain. The distinction between this category and the following one is that the arguments for the expected change should be significantly more compelling or likely than those for a contrary change. If the arguments tend towards a more equal footing, then level (ambiguous sign) is more appropriate.
5	Ambiguous sign or trend	Equally plausible contrary expectations	• In many cases it will not be possible to outline a definitive trend expectation.<br>• There may be plausible arguments for a change of sign or trend in either direction. If that is the case, state the opposing trends and outline the arguments on both sides.<br>• Note key uncertainties and assumptions in your arguments and how they may tip the balance in favour of one trend direction or the other.<br>• If information about the variable does not support this kind of supposition, then go to level 6.
6	Effective ignorance	Lacking or weakly plausible expectations	• Selecting this category does not mean that we know nothing about the variable. Rather, it means that our knowledge of the factors governing changes in the variable in the context of interest is so weak that we are effectively ignorant in this particular regard.<br>• If this category is selected, describe any expectations, such as they are, and note problems with them.

Supplementary Results

Relevance of health effects for adaptation policy

Temperature. A consistent line of argument in respondents' reasoning why *heat-related mortality* is most relevant for adaptation is that homes for the elderly, nursing homes, houses, and city/town planning in the Netherlands are completely not adapted to higher temperatures (and changes in temperatures). A participant makes this argument for Europe as a whole. Other arguments include: political interest, public perception, stress on the health care system, a lack of interest in the topic by the health care sector, and many people are at risk with potentially many victims in a short period of time. One expert notes that the high relevance score applies to the entire topic of heat-related mortality and disease.For *heat-related cardiovascular and respiratory problems*, respondents note that the effects could be substantial, and refer to many risk factors that could enhance the impact (traffic and city design and related air quality problems, and high incidence of obesity, cardiovascular disease and diabetes). For *dehydration*, a respondent notes again that homes for the elderly are not adapted. For heat-related stress and sleep disturbance notes that people would be tired during work/school (i.e. resulting in economic impacts).

Allergy. For *asthma*, respondents argue that the number of people already affected is already large, and rising, and is causing a considerable health burden. Changes herein due to climate change would add to this, resulting in high economic impacts (disease prevention, chronic disease treatment). For *hay fever (duration of pollen season)* a similar argument is made: a large number of people will be affected, and it could result in a loss of working days (also an economic impact). For *hay fever (pollen types/abundance/allergenicity)*, it is noted that effects could be substantial and difficult to adapt to.

Vector-borne diseases. For *endemic vector-borne diseases*, a respondent notes that a huge increase in disease risk has been observed in the past fifteen years, and that the costs for treatment of the chronic condition are high, as is the possible disease burden (e.g. due to neurological effects). For *incidents of non-endemic diseases*, respondents note that effects could be substantial and difficult to adapt to, and that incidents can be difficult to recognise and could result in public unrest. Similarly, for *epidemics of non-endemic diseases*, respondents stress a high potential health and economic impact, the link with public risk perception ('fright factors'), and stress on the health care system.

Food- and water-borne diseases. A respondent notes that the effects of *contamination of swimming/recreation water* could be substantial and difficult to adapt to. Another indicates that it is relevant because of the large amount of water in the Netherlands and recreational habits.

Air quality. Considering air quality-related health effects, one respondent notes for *respiratory problems due to ground-level ozone* that air pollution is already a considerable health problem and that climate change might add to this. Another indicates for *air quality-related cardiovascular problems* that effects could be substantial and difficult to adapt to.

Flooding and storm. Respondents who consider *flood-related mortality* to be relevant, indicate that flooding is a politically sensitive and culturally important topic for the Netherlands. The risk has a wide spatial extent and large potential impacts (e.g. spatial scale, societal 'signal value' of casualties). *Flood-related mental problems* are an underlying stress for populations in hazard areas and an under-recognised issue while effects have been reported even during evacuations (rather than only in case of actual flooding). *Flood-related exposure to dangerous substances and contaminants* could be relevant because there could be widespread exposure and it would be highly politically sensitive due to questions of blame.

UV. *UV-related skin cancer* could be relevant because of the cultural habits of sun bathing.

Uncertainty and options for adaptation

For health effects which respondents considered the most relevant, they were asked to indicate which policy options/strategies they considered to be particularly well-capable of dealing with the uncertainties associated with the effect – and which options/strategies would be very vulnerable to them. The answers will be discussed per health theme. No answers were provided for 'pests'. A total of 34 answers were provided, the majority (28) of which were made by 'adaptation and health' theme experts.

Temperature. Respondents suggested a diverse set of options for *heat-related mortality*, which would be capable of dealing with the uncertainties associated with this health effect. A number of respondents noted information supply and education as important, particularly aimed at vulnerable groups (e.g. the elderly) and other risk groups and caretakers of such groups. In any warning system for heat, responsibilities of relevant actors should be clear and the system should be based on scientific findings regarding risk conditions and options for adaptation. Respondents mention the need for action plans and contingency plans on what to do in case of heat several times. They refer to the Dutch National Heat Plan (VWS, 2007) in several instances. Aside from these 'soft' strategies, physical measures are mentioned as well. Planners could take heat into account in urban/area planning, e.g. by providing parks, open water, wind-corridors, et cetera. These could limit the effects of the urban heat island. Heat could also be (better) taken into consideration in building regulations, design and construction, for instance when developing homes for the elderly. One respondent also suggests further efforts on climate modelling. Regarding *heat-related cardiovascular and respiratory problems*, several options mentioned above are suggested again; for instance the National Heat Plan and area planning. Other suggestions include monitoring and surveillance, 'early warning', data collection, and development of models for scenario-analysis and impact assessment (i.e. more

research). In addition, a respondent notes that limiting/preventing summer smog is important. For dehydration, respondents refer again to the Heat Plan. Respondents did not suggest any options that were specifically vulnerable to the uncertainties associated with the theme of temperature-related effects.

Allergy. Regarding the theme of allergy, respondents suggested uncertainty-robust options for *asthma* and *hay fever (duration of pollen season, and pollen types/abundance/allergenicity).* Information supply and warning-systems – and related to this: better timing of medication intake – for hay fever patients are indicated as important by many participants. Furthermore, the allergenicity of the pollen that plants produce should be taken into account when selecting plants for public green spaces and nature management. Monitoring and surveillance, data collection, and development of models is useful as well. Medicine production and increases herein are mentioned as well. Respondents did not suggest any options that were specifically vulnerable to the uncertainties associated with this health theme.

Vector-borne diseases. Limiting the number of tick-bites and quick removal of ticks is important for limiting the consequences of climate change regarding *endemic vector-borne diseases*. Monitoring- and warning-systems are important as well. However, one respondent notes, risk communication and education are not always successful in reducing risky behaviour. Particular risk groups are people participating in outdoor recreation and rangers. For *incidents of non-endemic vector-borne diseases*, respondents suggest monitoring and surveillance to be important and uncertainty-robust. One respondent notes that education of health professionals on the topic of climate change is useful, as is the creation of flexible and generic action/contingency plans. Another again suggests early warning, data collection and model development. Furthermore, improving general hygiene, production of vaccines and medicines could be enhanced. Conversely, one participant indicates that the creation of large stockpiles of vaccines entails a large risk of overinvestment and is therefore a strategy that is vulnerable to uncertainty. A strategy such as pre-emptive vaccination could also entail the risk of negative health impacts or other side-effects (in addition to overinvestment risk). Action/contingency plans that are very (overly) specific for certain diseases and scenarios/transmission routes would be very vulnerable to surprises. Regarding *epidemics of non-endemic vector-borne diseases*, respondents note once more that monitoring and surveillance are uncertainty-robust. One respondent also suggests performing literature assessments and surveys on what is happening in other parts of the world regarding vector-borne diseases. Another indicates ‘early response’ and vaccination as possible options.

Food- and water-borne diseases. Information supply, monitoring/surveillance, early warning and data collection and model development are mentioned as options that are well-capable of dealing with the uncertainties. Other suggestions include good distribution of surface water, keeping in mind the link with urban design, and improving health care in general.

Air-quality. The effects of climate change on health via air quality can be reduced by measures which limit air pollution. Patients with respiratory conditions are a risk group. One respondent mentions once again: monitoring/surveillance, early warning, data collection and model development, keeping in mind the link with urban design, and better health care.

Flooding and storm. *Flood-related mortality* can be limited by improving water safety in general, via a combination of adaptation approaches that limit the probability and consequences of flooding. Good evacuation and monitoring strategies are also important. The two respondents who comment on this health effect both note that ‘hard engineering’ approaches are very vulnerable to uncertainties. They make risks more unpredictable and increase the vulnerability in case something does happen. *Flood-related mental health problems* can be reduced by keeping this issue in mind in disaster response and recovery plans, including in evacuation plans. It is also important to educate and train rescue workers, general practitioners and mental health professionals regarding this health aspect of flooding.

UV. Good information supply is suggested as a strategy that is well-capable of dealing with the uncertainties, for the effects of climate change via ultraviolet radiation.

Other. One respondent suggested that *societal disruption* of societal structures, possibly elsewhere, would have important consequences for health in the Netherlands He noted that present political trends all hamper adaptation and that societal change or transition is needed.
